# Supplementary material for: Conversion of Mixtures of Soybean Curd Residue and Kitchen Waste by Black Soldier Fly Larvae (Hermetia illucens L.)
Source: Insects. 2021 Dec 24;13(1):23. doi: 10.3390/insects13010023 (PMC8779397; doi:10.3390/insects13010023)
Supplement: Supplementary file 1 [file insects-13-00023-s001.zip › insects-1514824-supplementary.pdf]

Table S1. Crude protein and crude fat of BSFL fed on soybean curd residue and kitchen waste and their co-digestion mixtures.

Mixted organics	Larval protein content	Larval fat content	Other compounds
M0	44.17 ± 1.26^{ab}	32.71 ± 0.94^{c}	23.12 ± 0.68^{b}
M20	44.65 ± 1.68^{a}	34.76 ± 0.69^{ab}	20.59 ± 0.92^{d}
M30	44.50 ± 1.52^{a}	35.00 ± 1.35^{a}	20.50 ± 1.14^{d}
M40	43.15 ± 1.36^{b}	33.49 ± 1.12^{b}	23.36± 0.94^{b}
M50	43.27 ± 0.87^{b}	34.86 ± 0.98^{a}	21.87 ± 0.58^{c}
M100	42.91 ± 1.23^{c}	31.50 ± 0.67^{d}	25.59 ± 0.79^{a}

Six feed mixtures of SCR:KW were formulated: (0:100) M0, (20:80) M20, (30:70) M30, (40:60) M40, (50:50) M50, (100:0) M100. (Average ± SE; n = 3). Average values followed by the same letters within a column do not differ significantly ($p < 0.05$).
